# The associations between smartphone addiction and self-esteem, self-control, and social support among Chinese adolescents: A meta-analysis

**DOI:** 10.3389/fpsyg.2022.1029323

**Published:** 2022-11-07

**Authors:** Yueming Ding, Xiao Wan, Guangli Lu, Haitao Huang, Yipei Liang, Jingfen Yu, Chaoran Chen

**Affiliations:** ^1^Institute of Nursing and Health, School of Nursing and Health, Henan University, Kaifeng, China; ^2^Institute of Business Administration, School of Business, Henan University, Kaifeng, China

**Keywords:** smartphone addiction, self-esteem, self-control, social support, adolescent, meta-analysis

## Abstract

**Background:**

Smartphone addiction has become a social problem that affects the healthy growth of adolescents, and it is frequently reported to be correlated with self-esteem, self-control, and social support among adolescents.

**Methods:**

A meta-analysis was conducted by searching the PubMed, Web of Science, Embase, PsycINFO, PsycArticles, China National Knowledge Infrastructure (CNKI), WANFANG DATA, and Chongqing VIP Information Co., Ltd. (VIP) databases. Stata 16.0 was used to analyse the overall effect and test the moderating effect.

**Results:**

Fifty-six studies were included, involving a total of 42,300 participants. Adolescents' smartphone addiction had a moderately negative correlation with self-esteem (*r* = −0.25, 95% CI = −0.29 to −0.22, *p* < 0.001), a strong negative correlation with self-control (*r* = −0.48, 95% CI = −0.53 to −0.42, *p* < 0.001), and a weak negative correlation with social support (*r* = −0.16, 95% CI = −0.23 to −0.09, *p* < 0.001). Moderation analysis revealed that the correlation between adolescents' smartphone addiction and self-esteem was strongest when smartphone addiction was measured with the Mobile Phone Addiction Tendency Scale for College Students (MPATS; *r* = −0.38). The correlation between adolescents' smartphone addiction and self-control was strongest when self-control was measured with the Middle school students' Self-control Ability Questionnaire (MSAQ; *r* = −0.62). The effect of dissertations on smartphone addiction, self-control, and social support among adolescents was significantly larger than that of journal articles. The correlation between adolescents' smartphone addiction and social support was strongest when smartphone addiction was measured with the Mobile Phone Addiction Index (MPAI; *r* = −0.24). However, the correlations between adolescents' smartphone addiction and self-esteem, self-control, and social support were not affected by age or gender.

**Conclusion:**

There was a strong relationship between smartphone addiction and self-esteem, self-control, and social support among adolescents. In the future, longitudinal research should be carried out to better investigate the dynamic changes in therelationship between smartphone addiction and self-esteem, self-control, and social support.

**Systematic review registration:**

https://www.crd.york.ac.uk/PROSPERO/, identifier: CRD42022300061.

## Introduction

In the current, rapidly developing information age, smartphones have gradually become an indispensable tool in people's lives due to their characteristics of instant satisfaction, accessibility and function integration (Kuss et al., 2018; Noë et al., 2019; Recio-Rodriguez et al., 2019). The multiple functions of smartphones have brought various conveniences and benefits to adolescents' daily lives, but if individuals use smartphones excessively and uncontrollably for a long time, they may develop smartphone addiction (Cebi et al., 2019; Huang et al., 2022). Smartphone addiction (also known as “smartphone dependence,” “smartphone overuse,” or “problematic smartphone use”) is defined as a compulsive state in which an individual's physiological, psychological, and/or social functions are impaired due to the uncontrolled use of smartphones (Chóliz, 2010). It was categorized as a behavioral addiction (Takao et al., 2009; Yen et al., 2009), which manifests in symptoms including tolerance development and withdrawal, subjective loss of control, and functional impairment (Lee et al., 2014; Lin et al., 2016). At present, smartphone addiction has become a social problem that affects the healthy growth of adolescents. A large number of studies have found that smartphone addiction not only confers psychological and physiological effects on adolescents (e.g., anxiety, depression, and stress) but also negatively affects academic performance, coping styles, interpersonal relationships, etc., (Clayton et al., 2015; Samaha and Hawi, 2016; Lu et al., 2021; Diotaiuti et al., 2022; Wang et al., 2022; Yang et al., 2022; Zhang et al., 2022). Adolescents are in an important stage of developing peer relationships, pursuing autonomy and individuality, and changing behavior (Laursen and Hartl, 2013; Mak et al., 2014). Their strong curiosity and low level of self-control make them more vulnerable to problematic smartphone use (Munno et al., 2017), and thus, they have a higher risk of smartphone addiction (Cha and Seo, 2018; Kim and Lee, 2022). Data from multiple countries show that the incidence of smartphone addiction among adolescents has exceeded 30% (Davey and Davey, 2014; Lee and Lee, 2017; Xiang et al., 2019). Adolescence is a critical period of individual development (Lee C. P. et al., 2018) and an important period of development to reach psychophysiological maturity (Papalia et al., 2007). Therefore, it is necessary and urgent to explore the influencing factors and mechanism of smartphone addiction in adolescents to better prevent and control it.

A large number of studies have explored the influencing factors of smartphone addiction, among which self-esteem, self-control and social support are considered to be the three factors that are most closely related to smartphone addiction (Lee J. et al., 2018; Dou et al., 2020; Fu et al., 2020; Peng et al., 2020; Li et al., 2021). Self-esteem is a subjective evaluation of one's own ability, value and significance, which is conveyed by attitude and verbal behavior (Coopersmith, 1981). Adolescents with low self-esteem hold negative beliefs about the self and often have a low sense of safety and a low sense of identity in interpersonal communication (Passanisi et al., 2015). However, they have stronger desire for social recognition and respect (Cooper et al., 2017), and were more concerned with maintaining interpersonal relationships (Paz et al., 2017), and seem to prefer to technology-mediated communication (e.g., email; Joinson, 2004), which lead them to the massive use of the mobile phone to obtain reassurance in affective relationships (Billieux et al., 2015). Most studies support this view, namely, that self-esteem is negatively related to smartphone addiction. However, empirical findings on the strength of the association have been mixed. For example, Lee J. et al. (2018) found a strong negative correlation between self-esteem and smartphone addiction among adolescents (*r* = −0.35), Peng et al. (2020) found a moderately negative correlation between self-esteem and smartphone addiction among adolescents (*r* = −0.22), while Wang and Lei (2021) found a weak negative correlation between self-esteem and smartphone addiction among adolescents (*r* = −0.16).

Self-control refers to the ability of an individual to resist internal desires and external temptations to adhere to long-term goals (Tangney et al., 2004). The Deficient Self-regulation Model posits that adolescents with insufficient self-control may not be able to suppress their inner desire to use smartphones (Tokunaga and Rains, 2010), which may lead to an uncontrolled increase in smartphone use time and eventually to smartphone addiction. Many studies have revealed that self-control can negatively predict smartphone addiction. However, the correlation coefficients of different research results are quite different. For example, Li et al. (2016) found a strong negative correlation between self-control and smartphone addiction among adolescents (*r* = −0.49); Jeong et al. (2020) found a moderately negative correlation between self-control and smartphone addiction among adolescents (*r* = −0.29); and Li et al. (2021) found a weak negative correlation between self-control and smartphone addiction among adolescents (*r* = −0.07).

Social support is defined as the social support behaviors that individuals receive from other individuals and social networks (Heller et al., 1986). Compensatory Internet Use Theory suggests that when people encounter psychosocial problems in the real world, they may turn to the internet or smartphones to escape pain (Kardefelt-Winther, 2014). Adolescents with a low level of social support cannot establish intimate interpersonal relationships in real life, so they rely more on smartphones to meet their social needs, leading to a serious dependence on smartphones. Most studies supported this view and found a significant negative correlation between social support and smartphone addiction among adolescents (Fu et al., 2020). However, some researchers have argued the opposite view. For example, Jiao (2020) found a positive correlation between social support and smartphone addiction among adolescents (*r* = 0.13); Wang et al. (2018) found a nonsignificant correlation between social support and smartphone addiction among adolescents (*r* = 0.00).

To date, there is little consensus on the extent to which self-esteem, self-control and social support is correlated with smartphone addiction. Therefore, the first purpose of this study was to explore the relationship between adolescents' smartphone addiction and self-esteem, self-control, and social support.

As a secondary goal, we explored the potential moderators of effect sizes. Age, gender, publication type and measurement tools were considered as potential moderators. First, several previous meta-analyses have confirmed the age-specific distinctions in smartphone addiction (Zhang et al., 2020; Ran et al., 2022). Age may have some influence on the differences observed among different research samples. Second, compared with male adolescents, female adolescents tend to have lower levels of self-esteem (Estevez et al., 2017), their self-control is more vulnerable to external factors (Jo and Bouffard, 2014), and they receive more emotional support from others (Liebler and Sandefur, 2002). In addition, previous studies have revealed gender differences in the pattern of smartphone use (Jiang and Zhao, 2016; Volkmer and Lermer, 2019). Therefore, it is necessary to examine the moderating effect of gender. Third, in terms of publication type, studies with significant results are usually more likely to be published, so journal articles may exaggerate the real relationship between variables (Sterne et al., 2000). Finally, the focus of different measurement tools is different. The Mobile Phone Addiction Index (MPAI; Leung, 2008), Mobile Phone Addiction Tendency Scale for College Students (MPATS; Xiong et al., 2012), and Smartphone Addiction Scale (SAS; Kwon et al., 2013) are widely used tools for measuring smartphone addiction. These three measurement tools assess different aspects of smartphone addiction. The Rosenberg Self-Esteem Scale (RSES; Rosenberg, 1965) is widely used for measuring self-esteem. The scale assesses individuals' overall cognitive evaluation of themselves, while other measuring tools, such as the Adolescent Self-esteem Questionnaire (ASQ; Hafekost et al., 2016) assesses stressors related to adolescents' lives. The Self-control Scale (SCS; Tangney et al., 2004) and the Middle school students' Self-control Ability Questionnaire (MSAQ; Wang and Lu, 2004) are widely used tools for measuring self-control; the former is targeted toward college students and assesses two dimensions, i.e., cognition and behavior; the latter is targeted toward middle and high school students and assesses three dimensions, i.e., emotional self-control, behavior self-control and thinking self-control. In terms of tools for measuring social support, the Multidimensional Scale of Perceived Social Support (MSPSS; Zimet et al., 1988) assesses individuals' subjective feelings and evaluations of social support, while the Social Support Rating Scale (SSRS; Xiao and Yang, 1987) emphasizes not only individuals' subjective feelings and evaluations of social support, but also the investigation of objective support and the utilization degree of support.

## Methods

This meta-analysis followed the guidelines of Preferred Reporting Items for Systematic Reviews and Meta-Analyses (PRISMA; Page et al., 2021; see the checklist in Supplementary material 1) and was registered at PROSPERO (registration number CRD 42022300061).

### Literature search

The PubMed, Web of Science, Embase, PsycINFO, PsycArticles, China National Knowledge Infrastructure (CNKI), WANFANG DATA, and Chongqing VIP Information Co., Ltd. (VIP) databases were searched for eligible studies published up to July 28, 2022. To minimize publication bias, there were no restrictions regarding the date of publication. Search terms used for smartphones included “cell phone,” “mobile phone,” “smart phone,” “smartphone,” and “cellular phone.” Search terms used for addiction included “addiction,” “dependence,” “abuse,” “dependency,” “addicted to,” “overuse,” “problematic use,” and “compensatory use.” Search terms used for self-esteem included “self-esteem,” “Self-Esteem,” “self-concept,” “self-perception,” “Self-Perception,” “Self-Confidence,” and “self-respect.” Search terms used for self-control included “self-control,” “self-regulation,” “self-discipline,” “effortful-control,” and “impulse control.” Search terms used for social support included “social support,” “social care,” “online social support,” and “perceived social support.” A detailed search strategy is available in Supplementary material 2. Furthermore, the reference lists of the included studies were searched, and Chinese and English key words were used to identify additional eligible studies.

### Inclusion and exclusion criteria

The inclusion criteria were as follows: (a) the type of literature was a cross-sectional survey; (b) a validated tool was used to assess smartphone addiction and self-esteem, self-control, and social support; (c) the correlation coefficient between smartphone addiction and self-esteem, self-control, and social support was reported, and if the correlation coefficient of the total score was not reported, the full factor correlation coefficient should be reported; (d) the subjects were healthy adolescents; (e) published in English or Chinese; and (f) both published articles and dissertations were included. The exclusion criteria were as follows: (a) editorial, commentary, conference abstracts, and review articles; (b) studies with the same data published repeatedly; (c) literature with poor quality; and (d) studies with samples containing individuals with physical diseases or mental disorders.

### Data extraction

All studies were coded independently by two independent reviewers (YMD and XW). Any doubts or disagreements were resolved by consulting a third researcher (CRC). The following data were extracted: first author and year of publication, sample size, proportion of females, age, publication type, correlation coefficient, smartphone addiction scale, self-esteem scale, self-control scale, and social support scale (see Table 1). For the input of the correlation coefficient, there are the following coding standards: (a) If the correlation coefficient between smartphone addiction and self-esteem, self-control, and social support is not reported but the values of *F, T*, and χ^2^ are reported, they are transformed into the *r*-value by the corresponding formula (*r* = $\sqrt{\frac{t^{2}}{t^{2}+df}}$, *df* = n_1_ + n_2_-2; *r* = $\sqrt{\frac{F}{F+df_{e}}}$; *r* = $\sqrt{\frac{\chi^{2}}{\chi^{2}+N}}$) (Card, 2015). (b) The study effect size was encoded as an effect size according to the independent samples. If the study contained multiple independent samples, the article effect size was coded separately. (c) If only the correlation coefficients of certain dimensions between smartphone addiction and self-esteem, self-control, and social support were reported, the average of each dimension was taken before coding.

**Table 1:** Characteristics of the 56 studies included in the meta-analysis.

**First author (year)**	***N***	**Female %**	**Age**	**Publication type**	**SA measurement**	**Measurement instrument (Pearson's** ***r*****)**		
						**Self-esteem**	**Self-control**	**Social support**
Wang (2011)	664	58.1	3	D	Self-compiled	-	-	SSRS (−0.09)
Yu (2013)	484	60.3	2	D	WMPDS	SLCS-R (−0.27)	MSAQ (−0.54)	-
Xu and Bi (2014)	293	54.3	2	J	Self-compiled	-	-	SSRS (0.04)
Deng (2015)	1173	51.2	3	D	Self-compiled	-	MSAQ (-0.52)	-
Li et al. (2016)	913	53.5	3	J	MPATS	-	SCS (−0.49)	-
Pan (2016)	467	52.9	2	D	WMPDS	-	-	SSRS (−0.33)
Yang (2016)	403	54.8	3	D	MPAI	-	-	MSPSS (−0.41)
Wang et al. (2017)	768	56.0	2	J	SAS	RSES (−0.17)	-	-
Li (2017)	666	57.8	3	D	MPAI	-	MSAQ (−0.58)	-
Liu (2017)	533	53.3	3	D	DSDQ	-	SCS (−0.43)	PSSS (−0.13)
Jia (2018)	603	58.9	2	D	MPAI	RSES (−0.22)	-	-
Yu (2018)	1160	45.9	1	D	SAS-C	RSES (−0.21)	-	-
Liu (2018)	631	50.2	3	D	MPAI	-	MSAQ (−0.70)	-
Wang D. (2018)	958	49.8	2	D	WMPDS	-	MSAQ (−0.49)	-
Liu et al. (2018)	899	54.0	2	J	MPAI	-	DSCS (−0.39)	-
Duan (2018)	542	24.9	2	D	MPAI	-	-	SSRS (−0.14)
Wang et al. (2018)	655	45.0	2	J	SAS	-	-	MSPSS (0.00)
Wang Y. (2018)	1277	70.1	2	D	MPAI	-	-	SSRS (−0.10)
Zou (2018)	316	51.0	1	D	WMPDS	-	-	SSSUS (−0.04)
He (2019)	1075	44.7	3	J	MPATS	RSES (−0.36)	-	-
Li et al. (2019)	637	55.7	1	J	XSAI	RSES (−0.21)	-	-
Xiang et al. (2019)	643	52.7	3	J	SAS	-	SCS (−0.56)	-
Zheng (2019)	360	41.1	2	D	DSDQ	-	MSAQ (−0.51)	-
Zhu (2019)	407	50.9	1	D	WMPDS	-	MSAQ (−0.61)	-
Gao (2019)	447	1	2	D	MPAI	-	-	SSRS (−0.22)
Li (2019)	435	30.1	2	D	MPAI	-	-	SSRS (−0.13)
Wang et al. (2019)	772	56.0	3	J	SAS-SV	-	-	MSPSS (−0.03)
Gao (2020)	291	62.9	2	D	MPAI	RSES (−0.19)	-	-
Peng et al. (2020)	1912	63.2	3	J	MPAI	RSES (−0.22)	-	-
Li et al. (2020)	1102	51.5	3	J	MPPUS-10	-	ASCS (−0.55)	-
Xing (2020)	319	44.8	2	D	MPAI	-	MSAQ (−0.54)	-
Huang (2020)	736	51.6	1	D	MPATS	-	SCS (−0.46)	-
Ma (2020)	328	49.1	1	D	SAS-C	-	MSAQ (−0.65)	-
Gao et al. (2020) (sample 1)	642	46.4	1	J	MPAI	-	SCRC (−0.25)	-
Gao et al. (2020) (sample 2)	568	46.0	2	J	MPAI	-	SCRC (−0.32)	-
Ma et al. (2020)	981	48.5	1	J	MPAI	-	SCS (−0.34)	-
Niu et al. (2020)	726	48.6	3	J	MPAI	-	DSCS (−0.39)	-
Xiang et al. (2020)	947	48.4	3	J	SAS-SV	-	SCS (−0.54)	-
Jiao (2020)	373	54.8	2	D	MPATS	-	-	SSSUS (0.13)
Fu et al. (2020)	720	50.0	1	J	SAS-SV	-	-	MSPSS (−0.20)
Hu (2021)	413	52.1	1	D	MPAS	RSES (−0.30)	-	-
Liu et al. (2021)	697	44.5	2	J	MPAI	RSES (−0.32)	-	-
Yang (2021)	1138	66.5	2	D	MPAI	RSES (−0.25)	SCS (−0.40)	-
Kong et al. (2021)	1201	52.8	3	J	SQAPMPU	RSES (−0.33)	-	-
Wang and Lei (2021)	762	56.0	1	J	SAS-SV	RSES (−0.16)	-	-
Wang (2021)	850	48.0	3	D	MPAI	-	SCS (−0.62)	MSPSS (−0.36)
Zhao (2021)	844	51.5	2	D	MPAI	-	SCS (−0.71)	-
Li et al. (2021)	1034	38.8	3	J	MPAI	-	DMSC-S (−0.07)	-
Cui (2021)	924	53.9	2	D	MPAI		MSAQ (−0.29)	
Tian (2021)	2517	47.7	1	D	MPPUS-10			PSSS (−0.29)
Zhang (2021)	636	54.1	1	D	MPAI			PSSS (−0.30)
Li (2022)	420	65.7	3	J	MPAI	RSES (−0.28)		
Chen and Xiao (2022)	764	59.3	3	J	SAS-SV		SCS (−0.43)	
Tian et al. (2022)	620	44.2	1	J	MPAI		SCS (−0.50)	
Hu and Wang (2022)	926	46.4	3	J	SAS-SV		DSCS (−0.36)	
Wang and Jiang (2022)	728	52.6	2	J	CAS		SEL (−0.24)	

NR, Not Reported; 1, Middle school students; 2, High school students; 3, sample consisted of both middle school students and high school students; D, Dissertation; J, Journal; WMPDS, Wang's Mobile Phone Dependency Scale for Middle School Students; MPATS, Mobile Phone Addiction Tendency Scale for College Students; SAS, Smartphone Addiction Scale; MPAI, Mobile Phone Addiction Index; DSDQ, Deng's Smartphone Dependency Questionnaire for Middle School Students; SAS-C, Smartphone Addiction Scale for College Students; SAS-SV, Smartphone Addiction Scale-Short Version; K-SAS, Korean Smartphone Addiction Proneness Scale for Youth and Adults; IAT, the modified version of Internet Addiction Test; XSAI, Xie's Smartphone Addiction Inventory; LSDI, Lee's Smartphone Dependency Instrument; SABAS, Smartphone Application-Based Addiction Scale; MPPUS-10, a short version of the Mobile Phone Problem Use Scale; COS, Cell-Phone Over-Use Scale; MPAS, Mobile Phone Addiction Scale; SQAPMPU, Self-rating Questionnaire for Adolescent Problematic Mobile Phone Use; SLCS-R, Self-Liking/Self-Competence Scale-Revised; RSES, Rosenberg Self-Esteem Scale; LSES, the Lifespan Self-Esteem Scale; CSEC = Coopersmith Self-Esteem Scale; ISLQ, Inventory of Subjective Life Quality; ASQ, Adolescent Self-esteem Questionnaire; MSAQ, the Middle school students' Self-control Ability Questionnaire; SCS, Self-Control Scale; K-SCS, Korean Self Control Scale; DSCS, Dong's Self-control Scale; BSCS, Brief Self Control Scale; ASCS, Adolescent Self-control Scale; SCRC, Self-Control Rating Scale; GF-SRS, Goal-Focused Self-Regulation Scale; DMSC-S, Dual-Mode of Self-Control Scale; SSRS, Social Support Rating Scale; MSPSS, Multidimensional Scale of Perceived Social Support; PSSS, Perceived Social Support Scale; SSSUS, Social Support Scale for University Students.

### Quality assessment

The quality of the studies was assessed independently by two reviewers (YMD and GLL). Any doubts or disagreements were resolved by centralized discussion (at least three people) or by consulting a third researcher (CRC). The methodological quality of the included studies was assessed by using the nine-item Joanna Briggs Institution Critical Appraisal Checklist for Studies Reporting Prevalence Data (Munn et al., 2015). The score for each item is zero (“no,” “unclear,” or “not applicable”) or one (“Yes”), and the highest score is nine. Higher scores reflected better methodological quality.

### Statistical analysis

Stata 16.0 was used for meta-analysis, and effect sizes were calculated as correlations (*r*) in this study. Specifically, the correlations (*r*) were first converted to the corresponding Fisher's *Z*-value by using the Fisher transform, weighted based on the sample size with 95% confidence intervals: *Z* = 0.5^*^ln[ (1+r)/(1–r)], where the variance of *Z* is V_*Z*_ = 1/n−3 and the standard deviation of *Z* is SE_*Z*_ = square root of (1/n−3). The degree of association was interpreted through Gignac and Szodorai's criteria (Gignac and Szodorai, 2016) with effects of 0.10 deemed small, 0.20 deemed moderate, and equal to and larger than 0.30 interpreted as high. Moreover, we used meta-regression analysis for continuous moderators and subgroup analysis for categorical moderators. Publication bias was analyzed by funnel plots and Egger's linear regression test, and Cochran's *Q* and *I*^2^ statistics were used to assess heterogeneity. When the *Q* value was significant (*p* < 0.1) and *I*^2^ ≥ 50%, this indicated a heterogeneity in the study, and thus, the random effects model was used; otherwise, the fixed effects model was chosen (Higgins and Green, 2008). In addition, subgroup analysis was conducted to investigate the sources of heterogeneity.

## Results

### Study selection

**Figure 1:**
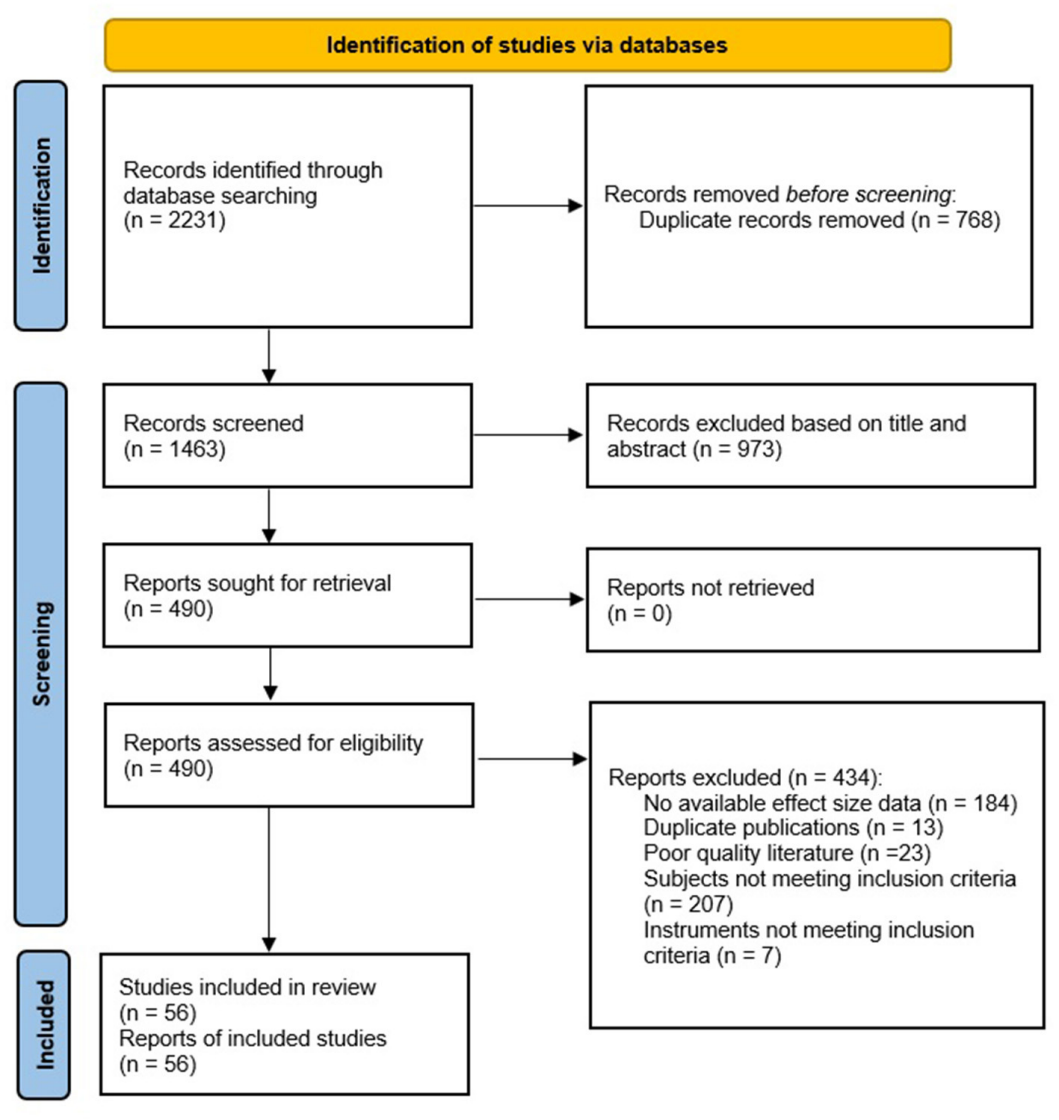
The flow chart of the study selection process.

The initial search yielded 2,231 studies. After duplicate records (*n* = 768) were removed and 973 studies were excluded on the basis of title and abstract, the full texts of 490 papers were reviewed. A total of 434 studies were excluded for various reasons (listed in Figure 1). A total of 56 studies met the inclusion criteria and were included in the meta-analysis.

### Characteristics of the included studies and quality assessment

Fifty-six studies were included in the meta-analysis, which were published between 2011 and 2022. Collectively, 42,300 participants were enrolled in the included studies, and they were all recruited from schools, with sample sizes ranging from 291 to 2,517. Of the 41,645 participants whose gender was reported, 51.2% were female. The MPAI was the most frequently used tool to assess smartphone addiction status among participants (*n* = 27); the RSES was the most frequently used tool to assess self-esteem (*n* = 13); the SCS was the most frequently used tool to assess self-control (*n* = 11); and the SSRS was the most frequently used tool to assess social support (*n* = 7; see Table 1). Overall, the quality of the included studies was at a medium or high level (total score ≥ 6). Detailed information about the quality assessment of each study can be found in Supplementary material 3.

### Effect size and heterogeneity test

A heterogeneity test was conducted on the included effect sizes, and the results showed that the Q values of self-esteem, self-control and social support were 49.96 (*p* < 0.001), 759.02 (*p* < 0.001), and 231.91 (*p* < 0.001), respectively, and the *I*^2^-values were 74.0, 96.3, and 93.1%, respectively. These *I*^2^-values were higher than the 50% rule proposed by Higgins et al. (2003), indicating a high level of heterogeneity among studies. Therefore, the random effects model was selected for meta-analysis. The results also show that it is necessary to explore the moderating variables that affect the relationship between them.

The random effects model showed that adolescents' smartphone addiction was moderately negatively correlated with self-esteem, strongly negatively correlated with self-control, and weakly negatively correlated with social support (self-esteem: *r* = −0.25, 95% CI = −0.29 to −0.22, *p* < 0.001; self-control: *r* = −0.48, 95% CI = −0.53 to −0.42, *p* < 0.001; social support: *r* = −0.16, 95% CI = −0.23 to −0.09, *p* < 0.001; Table 2).

**Table 2:** Effect size and its heterogeneity test and publication bias test.

**Outcome variable**	***k***	***N***	***r***	**95% CI for *r***	**Heterogeneity test**			**Publication bias test**			
					***Q***	***df***	***I^2^***	**Egger's intercept**	***SE***	**95% CI**	***p***
Self-esteem	14	11,561	−0.25	[−0.29, −0.22]	49.96^***^	13	74.0%	0.28	2.24	[−4.61, 5.16]	0.90
Self-control	29	21,844	−0.48	[−0.53, −0.42]	759.02^***^	28	96.3%	−7.66	5.72	[−19.40, 4.07]	0.19
Social support	17	11,900	−0.16	[−0.23, −0.09]	231.91^***^	16	93.1%	4.86	3.03	[−1.61, 11.32]	0.13

*** *p* < 0.001.

### Moderation analysis

The heterogeneity of effects across studies was explored through moderation analysis. Subgroup analysis and meta-regression analysis were used to examine the moderating effects of categorical variables (age, publication type, tool for measuring smartphone addiction, tool for measuring self-esteem, tool for measuring self-control and tool for measuring social support) and continuous variables (gender), respectively.

As shown in Tables 3, 4, the tool for measuring smartphone addiction significantly moderated the relationship between smartphone addiction and self-esteem (*p* < 0.001). In terms of the tool for measuring smartphone addiction, the correlation was strongest when the MPATS was used (*r* = −0.38, 95% CI = −0.44 to −0.32), followed by the use of other scales (*r* = −0.27, 95% CI = −0.33 to −0.21), the MPAI (*r* = −0.25, 95% CI = −0.29 to −0.22), and the SAS (*r* = −0.17, 95% CI = −0.22 to −0.12). However, the moderating effects of age, gender, publication type and tool for measuring self-esteem on smartphone addiction and self-esteem were not significant (all *p* > 0.05).

**Table 3:** Subgroup analyses of the summary correlation between smartphone addiction and self-esteem.

**Moderators**	***k***	***N***	***r***	**95% CI**	**Between-group effect (*Q_*BET*_*)**	***I^2^ (%)***	***p***
**Age**					0.59		0.443
Middle school students	4	2,972	−0.22	[−0.27, −0.17]		48.6	
High school students	6	3,981	−0.25	[−0.29, −0.20]		56.0	
**Article type**					0.43		0.511
Dissertation	6	4,089	−0.24	[−0.27, −0.21]		0.0	
Journal	8	7,472	−0.26	[−0.32, −0.21]		84.2	
**SA measurement**					28.09^***^		0.000
MPATS	1	1,075	−0.38	[−0.44, −0.32]		0.0	
MPAI	6	5,061	−0.25	[−0.29, −0.22]		40.6	
SAS/SAS-SV	2	1,530	−0.17	[−0.22, −0.12]		0.0	
Others	5	3,895	−0.27	[−0.33, −0.21]		68.4	
**Self-esteem measurement**					0.12		0.724
RSES	13	11,077	−0.26	[−0.30, −0.22]		0.0	
Others	1	484	−0.27	[−0.36, −0.18]		75.9	

*** *p* < 0.001.

**Table 4:** Univariate regression analysis of continuous variables (random effect model).

**Moderators**		***k***	***SE***	***t***	**95% CI**	***p***
Female (%)	Self-esteem	14	0.24	1.55	[−0.15, 0.89]	0.15
	Self-control	29	0.01	−0.86	[−0.02, 0.01]	0.40
	Social support	17	0.26	−0.19	[−0.60, 0.50]	0.85

The publication type and the tool for measuring self-control significantly moderated the relationship between smartphone addiction and self-control (*p* < 0.01 and *p* < 0.01, respectively). In terms of publication type, the correlation for dissertations (*r* = −0.61, 95% CI = −0.70 to −0.52) was significantly stronger than that for journal articles (*r* = −0.42, 95% CI = −0.51 to −0.33). In the tool for measuring self-control, the correlation was strongest when self-control was measured with MSAQ (*r* = −0.62, 95% CI = −0.72 to −0.51), followed by the SCS (*r* = −0.56, 95% CI = −0.65 to −0.46) and other scales (*r* = −0.34, 95% CI = −0.46 to −0.22). However, age, gender and the tool for measuring smartphone addiction did not moderate the relationship between smartphone addiction and self-control (all *p* > 0.05; Tables 4, 5).

**Table 5:** Subgroup analyses of the summary correlation between smartphone addiction and self-control.

**Moderators**	***k***	***N***	***r***	**95% CI**	**Between-group effect (*Q_*BET*_*)**	***I^2^ (%)***	***p***
**Age**					1.76		0.185
Middle school students	7	4,558	−0.57	[−0.75, −0.40]		97.3	
High school students	9	6,378	−0.44	[−0.52, −0.36]		90.7	
**Article type**					9.02^**^		0.003
Dissertation	15	10,351	−0.61	[−0.70, −0.52]		95.2	
Journal	14	11,493	−0.42	[−0.51, −0.33]		95.7	
**SA measurement**					0.98		0.806
MPATS	2	1,649	−0.52	[−0.57, −0.47]		0.0	
MPAI	14	10,842	−0.49	[−0.62, −0.36]		97.8	
SAS/SAS-SV	4	3,280	−0.52	[−0.64, −0.40]		91.7	
Others	9	6,073	−0.56	[−0.66, −0.47]		92.5	
**Self-control measurement**					12.10^**^		0.002
SCS	11	8,969	−0.56	[−0.65, −0.46]		95.0	
MSAQ	10	6,250	−0.62	[−0.72, −0.51]		94.0	
Others	8	6,625	−0.34	[−0.46, −0.22]		96.1	

** *p* < 0.01.

The publication type and the tool for measuring smartphone addiction significantly moderated the relationship between smartphone addiction and social support (*p* < 0.05 and *p* < 0.001, respectively). In terms of publication type, the correlation for dissertations (*r* = −0.19, 95% CI = −0.27 to −0.12) was significantly stronger than that for journal articles (*r* = −0.05, 95% CI = −0.16 to 0.05). In terms of the tool for measuring smartphone addiction, the correlation was strongest when smartphone addiction was measured with MPAI (*r* = −0.24, 95% CI = −0.34 to −0.14), followed by the other scales (*r* = −0.15, 95% CI = −0.27 to −0.03), MPATS (*r* = 0.13, 95% CI = 0.03 to 0.23), and the SAS (*r* = −0.08, 95% CI = −0.20 to 0.05). However, age, gender, publication type, and tool for measuring social support did not differ between subgroups (all *p* > 0.05; Tables 4, 6).

**Table 6:** Subgroup analyses of the summary correlation between smartphone addiction and social support.

**Moderators**	***k***	***N***	***r***	**95% CI**	**Between-group effect (*Q_*BET*_*)**	***I^2^ (%)***	***p***
**Age**					3.45		0.063
Middle school students	4	4,189	−0.22	[−0.32, −0.13]		86.9	
High school students	8	4,489	−0.10	[−0.19, −0.00]		89.6	
**Article type**					4.45^*^		0.035
Dissertation	13	9,460	−0.19	[−0.27, −0.12]		92.7	
Journal	4	2,440	−0.05	[−0.16, 0.05]		85.4	
**SA measurement**					27.65^***^		0.000
MPATS	1	373	0.13	[0.03, 0.23]		0.0	
MPAI	7	4,590	−0.24	[−0.34, −0.14]		91.5	
SAS/SAS-SV	3	2,147	−0.08	[−0.20, 0.05]		88.2	
Others	6	4,790	−0.15	[−0.27, −0.03]		92.7	
**Social support measurement**					0.54		0.763
SSRS	7	4,125	−0.14	[−0.22, −0.06]		83.2	
MSPSS	5	3,400	−0.21	[−0.38, −0.04]		95.9	
Others	5	4,375	−0.14	[−0.29, 0.02]		95.1	

* *p* < 0.05.

*** *p* < 0.001.

### Publication bias

**Figure 2:**
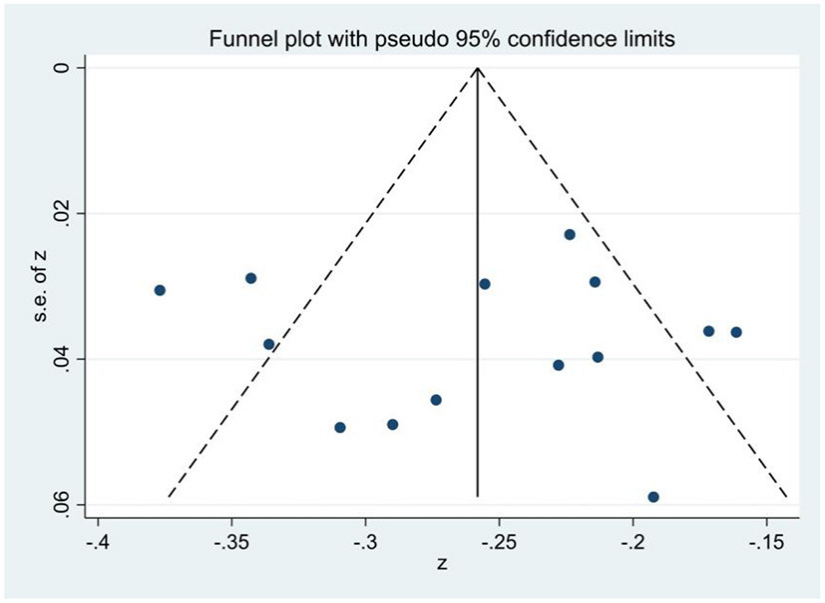
Funnel plot of the correlation of smartphone addiction and self-esteem.

**Figure 3:**
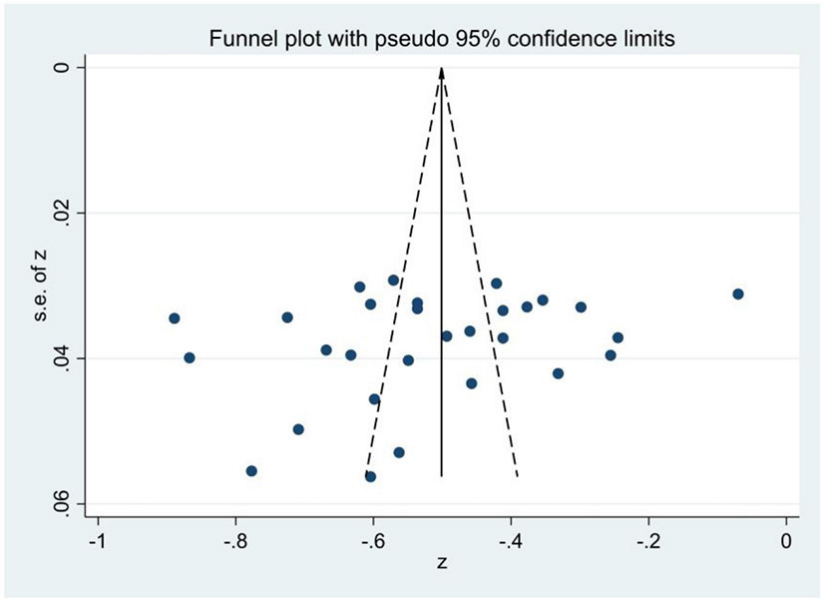
Funnel plot of the correlation of smartphone addiction and self-control.

**Figure 4:**
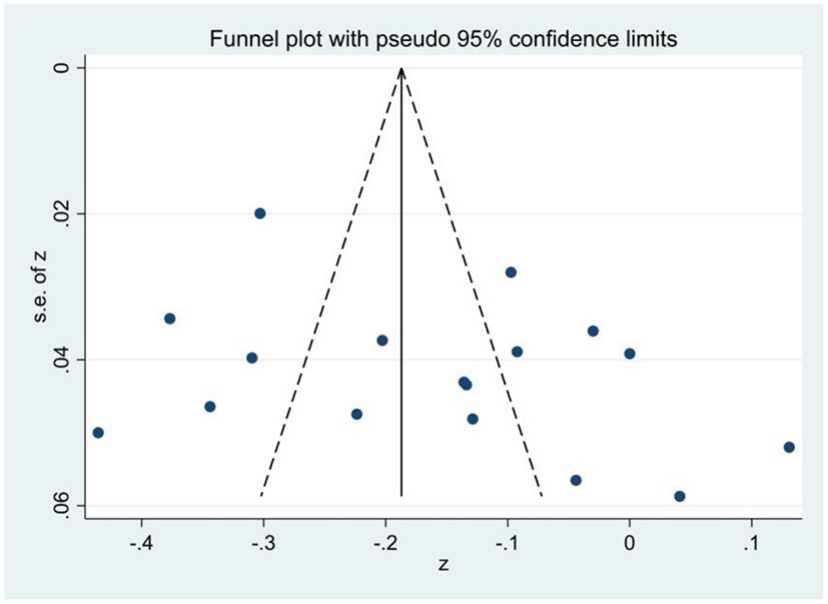
Funnel plot of the correlation of smartphone addiction and social support.

Publication bias was detected using a funnel plot and Egger's linear regression test. First, Figures 2–4 showed that the effect sizes of the relationship between smartphone addiction and self-esteem, self-control, and social support were basically evenly distributed on both sides of the overall effect sizes, indicating that the risk of publication bias was small in the study. Second, Egger's linear regression tests found that the *p*-values of self-esteem (*p* = 0.90), self-control (*p* = 0.19), and social support (*p* = 0.13) were all >0.05, which further indicated that there was no publication bias in this study, and the estimated results of meta-analysis were relatively reliable (Table 2).

### Sensitivity analysis

**Figure 5:**
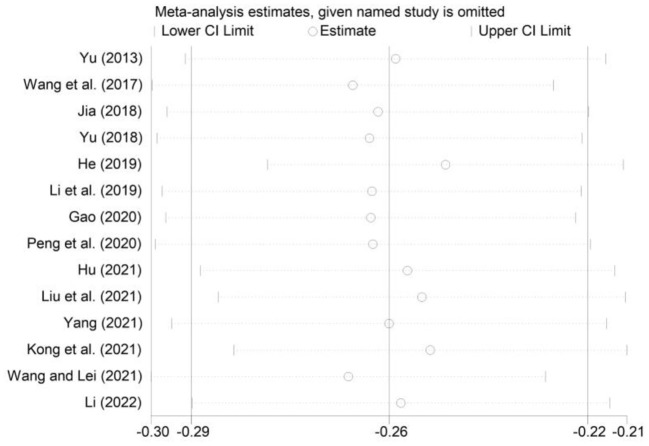
Sensitivity analysis of the correlation between smartphone addiction and self-esteem.

**Figure 6:**
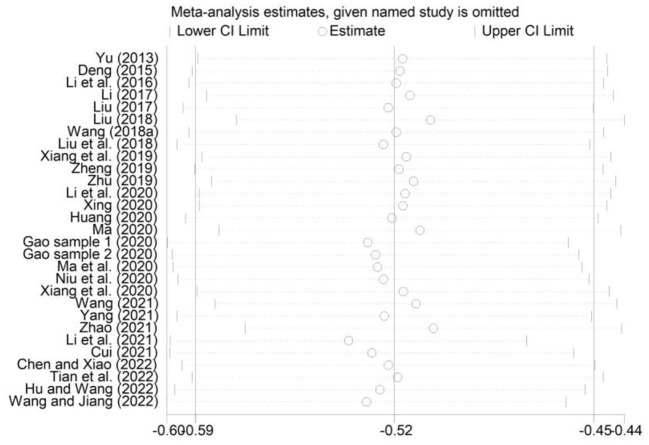
Sensitivity analysis of the correlation between smartphone addiction and self-control.

**Figure 7:**
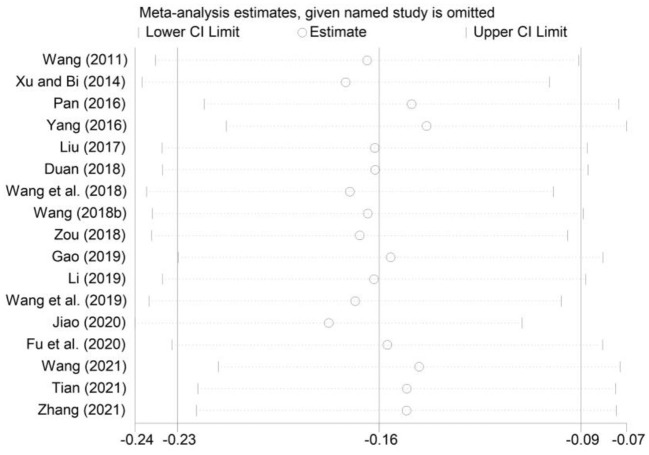
Sensitivity analysis of the correlation between smartphone addiction and social support.

To evaluate the robustness of our findings, we used the one-by-one elimination method for sensitivity analysis. As shown in Figures 5–7, the effect size after removing the studies one at a time is within the 95% CI value of the total effect size. Overall, the results were not significantly changed, suggesting that the results of this study were relatively stable.

## Discussion

### Relationship between smartphone addiction and self-esteem, self-control, and social support

This study clarifies the disagreement over the magnitude of the relationship between self-esteem, self-control and smartphone addiction and the magnitude and direction of the relationship between social support and smartphone addiction. The details were as follows. First, the results showed that adolescents' smartphone addiction had a moderately negative correlation with self-esteem (*r* = −0.25, *p* < 0.001), indicating that with the decrease of self-esteem, smartphone addiction is more likely to occur, which is consistent with the conclusions of most previous studies. Yuchang et al. (2017) found that adolescents with low self-esteem are often at a disadvantage in social interactions and receive less social support, and they are more likely to feel extremely lonely; thus, they are more likely to develop smartphone addiction. You et al. (2019) found that adolescents with low self-esteem usually have cognitive distortions and maladaptive emotional regulation (Billieux, 2012), which leads to higher social anxiety, and have to overuse smartphones to obtain reassurance in affective relationships. Therefore, educators should pay attention to strengthening the improvement of adolescents' self-esteem. For example, group-assisted activities can not only improve the relationship between adolescents but also improve adolescents' self-cognition level in interpersonal communication to intervene in the formation of smartphone addiction.

Second, the results showed that adolescents' smartphone addiction had a strong negative correlation with self-control (*r* = −0.48, *p* < 0.001), indicating that adolescents with low self-control are more likely to be addicted to smartphones, which is consistent with the conclusions of most previous studies. Li et al. (2021) found that adolescents with lower self-control are at higher risk of developing smartphone addiction due to their escapist thoughts. Jiang and Zhao (2016) found that the short-term pleasure and satisfaction benefiting from the chat and shopping functions of smartphones to adolescents with low self-control will increase the likelihood of smartphone overuse. This suggest that educators should prioritize enhancing adolescents' self-control level when conducting smartphone addiction interventions. Group cognitive-behavioral therapy (Zeidi et al., 2020) and maintaining a regular academic study program (Oaten and Cheng, 2006) may be effective ways to improve self-control ability and help to reduce the possibility of adolescents' smartphone addiction.

Moreover, the results showed that adolescents' smartphone addiction had a weak negative correlation with social support (*r* = −0.16, *p* < 0.001), indicating that adolescents with low social support are prone to smartphone addiction, which is consistent with the conclusions of most previous studies (Li, 2019; Fu et al., 2020). Additionally, these results reject the view that there is a positive correlation between social support and smartphone addiction (Jiao, 2020), and reject the view that there is no significant association between social support and smartphone addiction (Wang et al., 2018; Zou, 2018). Furthermore, these results imply that to effectively prevent and reduce smartphone addiction among adolescents, it is necessary to establish a good social support system. It is worth noting that according to the cognitive-behavioral model of Davis (2001), an individual's addictive behavior is not entirely due to the lack of realistic social support but rather is due to individuals being unaware of the existing social support and thus being unable to make good use of the existing social support. Therefore, in addition to giving adolescents sufficient instrumental social support, attention should also be devoted to improving the level of adolescents' emotional social support and the utilization degree of support.

### Moderating effects

Publication type significantly moderated the relationship between adolescents' smartphone addiction, self-control, and social support. The effect of dissertations is significantly stronger than that of journal articles. This finding is inconsistent with previous studies. Generally, in meta-analysis studies with publication bias, the effect of journal articles is larger than that of dissertations (Pan et al., 2020). This difference may be related to the quality of the studies and the rigor of the review.

The tools for measuring smartphone addiction significantly moderated the relationship between adolescents' smartphone addiction and both self-esteem and social support. First, in terms of self-esteem, the MPATS (Xiong et al., 2012; *r* = −0.38) had the strongest effect. This may be due to the different perspectives of the MPATS and other scales. The MPATS is more based on the subjective experience of smartphone users' inner processing activities and social interaction. According to the sociometer theory, self-esteem is a measure of the state of social relationship status. Adolescents with low self-esteem show high social anxiety and interpersonal sensitivity (Leary et al., 1995), which makes it difficult for them to establish good interpersonal relations in the real world and have the psychological tendency of escapism, thus having a high level of smartphone addiction. Second, in terms of social support, the MPAI (*r* = −0.24) had the strongest effect. The reason may be that the MPAI mainly focuses on describing the impact of smartphones on users' behavior and impairment of social functions. Studies have shown that adolescents using smartphones as a substitute for their contact with society will have lower levels of social functioning (Mynatt et al., 1998), and when the social support needs of adolescents cannot be met in reality, they will use smartphones to reduce the negative psychological effects of social exclusion (Schick et al., 2018), which further increases the possibility of smartphone addiction, so MPAI showed a stronger correlation.

The tools for measuring self-control significantly moderated the relationship between adolescents' smartphone addiction and self-control. The MSAQ (Wang and Lu, 2004) had the strongest effect size, followed by the SCS (Tangney et al., 2004) and the other scales. The reason may be due to the different perspectives of different measurement instruments. The MSAQ is applicable to adolescents, while the SCS and other scales are mainly applicable to college students. In comparison, the MSAQ scale is more targeted toward the subjects of the current study (adolescents). Studies have shown that adolescents have lower levels of self-control than college students, and they are more prone to problematic behaviors, such as smartphone addiction (Chambers et al., 2003; Lopez-Fernandez et al., 2014; Kiss et al., 2020). Therefore, the use of the MSAQ showed a stronger effect.

### Limitations and prospects

Previous studies on the relationship between smartphone addiction and self-esteem, self-control, and social support among adolescents have been inconsistent. In this study, the meta-analysis was used to investigate the relationship between smartphone addiction and self-esteem, self-control, and social support among adolescents, and to clarify the controversy about the size of the correlation between them in the empirical study. However, this study also has some limitations. First, the data of this study were collected through a questionnaire survey, so information bias and reporting bias are inevitable, and more objective forms of data collection can be considered for future research. Second, the studies included in this meta-analysis mainly focused on adolescents. In the future, the subject group can be further expanded to explore whether there are differences in the relationship between smartphone addiction and self-esteem, self-control, and social support among different subject groups. Finally, the studies retrieved in this meta-analysis were all cross-sectional studies. Whether there is a causal relationship between the relevant factors found and smartphone addiction needs to be further verified by longitudinal studies in the future.

### Implications

This study is of great significance for the prevention and intervention of adolescents' smartphone addiction. First, the results describe the correlation between adolescents' smartphone addiction and self-esteem, self-control and social support, which can provide a reference for future studies. Additionally, this means that attaching great importance to the improvement of self-esteem, self-control, and social support may important for reducing the occurrence of smartphone addiction among adolescents. Second, there was no significant difference between age and genders in the problems of smartphone addiction accompanied by low self-esteem, low self-control and low social support. In future interventions, it will be important to pay attention to the comprehensiveness of group of adolescents of different ages and genders coverage. Third, the measurement tool of smartphone addiction significantly moderated the relationship between adolescents' smartphone addiction, self-esteem and social support. This reminds researchers and clinicians to use common criteria to define smartphone addiction whenever possible to reduce potential differences. Finally, there are differences in the predictive power obtained by using different self-control measurement tools, which reminds researchers that they should choose appropriate self-control measurement tools according to the purpose and object of their own research as much as possible.

## Conclusion

The current meta-analysis found that adolescent smartphone addiction was moderately negatively associated with self-esteem, had a strong negative correlation with self-control, and had a weak negative correlation with social support, indicating that adolescents with low levels of self-esteem, self-control and social support were more likely to develop smartphone addiction. Therefore, in the prevention and intervention of smartphone addiction among adolescents, more attention should be given to adolescents with low levels of self-esteem, self-control and social support. Not only should sufficient social support be given to meet their psychological needs, but also to help them improve their self-esteem and self-control in daily life and study, learn to use smartphones reasonably and avoid the harm of addiction.

## Data availability statement

The original contributions presented in the study are included in the article/Supplementary material, further inquiries can be directed to the corresponding authors.

## Author contributions

YD and XW: study design and drafting of the manuscript. YD, CC, GL, HH, YL, and JY: analysis and interpretation of data and critical revision of the manuscript. GL and CC: data curation and supervision. All authors approved the final manuscript to be published.

## Funding

This research was funded by the Graduate Education Innovation and Quality Improvement Program of Henan University (grant number SYL19060141), the Henan Provincial Social Science Planning Decision Consulting Project (grant number 2018JC38), the Graduate Education Reform and Quality Improvement Project of Henan Province (grant number YJS2021AL074), the Key Program of Research and Practice on Undergraduate Teaching Reform of Henan University (grant number HDXJJG2020-25), and the Survey Subject of Henan Federation of Social Sciences Circles-Research on the Status Quo and Cultivation Mechanism of Social and Emotional Abilities of Youth in Henan Province (grant number SKL-2022-55).

## Conflict of interest

The authors declare that the research was conducted in the absence of any commercial or financial relationships that could be construed as a potential conflict of interest.

## Publisher's note

All claims expressed in this article are solely those of the authors and do not necessarily represent those of their affiliated organizations, or those of the publisher, the editors and the reviewers. Any product that may be evaluated in this article, or claim that may be made by its manufacturer, is not guaranteed or endorsed by the publisher.
