# Supplementary material for: The associations between smartphone addiction and self-esteem, self-control, and social support among Chinese adolescents: A meta-analysis
Source: Front Psychol. 2022 Nov 7;13:1029323. doi: 10.3389/fpsyg.2022.1029323 (PMC9677120; doi:10.3389/fpsyg.2022.1029323)
Supplement: Supplementary file 2 [file Table_2.DOCX]

**Search strategy**

***PubMed***

#1: “Cell Phone”[Mesh]

#2: (((mobile phone[Title/Abstract]) OR (smart phone[Title/Abstract])) OR (smartphone[Title/Abstract])) OR (cellular phone[Title/Abstract])

#3: #1 or #2

#4: “Behavior, Addictive”[Mesh]

#5: (((((((addiction[Title/Abstract]) OR (dependence[Title/Abstract])) OR (abuse[Title/Abstract])) OR (dependency[Title/Abstract])) OR (addicted to[Title/Abstract])) OR (overuse[Title/Abstract])) OR (problem use[Title/Abstract])) OR (compensatory use[Title/Abstract])

#6: #4 or #5

#7: “Self Concept”[Mesh]

#8: ((((((self-esteem[Title/Abstract]) OR (Self Esteem[Title/Abstract])) OR (self-concept[Title/Abstract])) OR (self-perception[Title/Abstract])) OR (Self Perception[Title/Abstract])) OR (Self Confidence[Title/Abstract])) OR (self-respect[Title/Abstract])

#9: #7 or #8

#10: “Self-Control”[Mesh]

#11: (((self-regulation[Title/Abstract]) OR (self-discipline[Title/Abstract])) OR (effortful-control[Title/Abstract])) OR (impulse control[Title/Abstract])

#12: #10 or #11

#13: “Social Support”[Mesh]

#14: (((social care[Title/Abstract])) OR (online social support[Title/Abstract])) OR (perceived social support[Title/Abstract])

#15: #13 or #14

#16: #3 and #6 and #9

#17: #3 and #6 and #12

#14: #3 and #6 and #15

***Web of science***

TS=(“cell Phone” OR “mobile phone” OR “smart phone” OR smartphone OR “cellular phone”) AND TS=(addiction OR dependence OR abuse OR dependency OR “addicted to” OR overuse OR “problem use” OR “compensatory use”) AND TS=(self-esteem OR “Self Esteem” OR self-concept OR self-perception OR “Self Perception” OR “Self Confidence” OR self-respect)

Timespan: All years

TS=(“cell Phone” OR “mobile phone” OR “smart phone” OR smartphone OR “cellular phone”) AND TS=(addiction OR dependence OR abuse OR dependency OR “addicted to” OR overuse OR “problem use” OR “compensatory use”) AND TS=(self-control OR self-regulation OR self-discipline OR effortful-control OR “impulse control”)

Timespan: All years

TS=(“cell Phone” OR “mobile phone” OR “smart phone” OR smartphone OR “cellular phone”) AND TS=(addiction OR dependence OR abuse OR dependency OR “addicted to” OR overuse OR “problem use” OR “compensatory use”) AND TS=(“social support” OR “social care” OR “online social support” OR “perceived social support”)

Timespan: All years

***Embase***

#1. 'mobile phone'/exp

#2. 'cell phone':ab,ti

#3. 'smart phone':ab,ti

#4. 'smartphone':ab,ti

#5. 'cellular phone':ab,ti

#6. #1 OR #2 OR #3 OR #4 OR #5

#7. 'addiction'/exp

#8. 'dependence':ab,ti

#9. 'abuse':ab,ti

#10. 'dependency':ab,ti

#11. 'addicted to':ab,ti

#12. 'overuse':ab,ti

#13. 'problem use':ab,ti

#14. 'compensatory use':ab,ti

#15. #7 OR #8 OR #9 OR #10 OR #11 OR #12 OR #13 OR #14

#16. 'self esteem'/exp

#17. 'self-esteem':ab,ti

#18. 'self-concept':ab,ti

#19. 'self-perception':ab,ti

#20. 'self perception':ab,ti

#21. 'self confidence':ab,ti

#22. 'self-respect':ab,ti

#23. #16 OR #17 OR #18 OR #19 OR #20 OR #21 OR #22

#24. #6 AND #15 AND #23

#25. 'self control'/exp

#26. 'self-control':ab,ti

#27. 'self-regulation':ab,ti

#28. 'self-discipline':ab,ti

#29. 'effortful-control':ab,ti

#30. 'impulse control':ab,ti

#31. #25 OR #26 OR #27 OR #28 OR #29 OR #30

#32. #6 AND #15 AND #31

#33. 'social support'/exp

#34. 'social care':ab,ti

#35. 'online social support':ab,ti

#36. 'perceived social support':ab,ti

#37. #33 OR #34 OR #35 OR #36

#38. #6 AND #15 AND #37

***PsycINFO and PsycArticles***

Boolean/Phrase: (“cell Phone” OR “mobile phone” OR “smart phone” OR smartphone OR “cellular phone”) AND (addiction OR dependence OR abuse OR dependency OR “addicted to” OR overuse OR “problem use” OR “compensatory use”) AND (self-esteem OR “Self Esteem” OR self-concept OR self-perception OR “Self Perception” OR “Self Confidence” OR self-respect)

Limiters: Language – English

Timespan: All years

Boolean/Phrase: (“cell Phone” OR “mobile phone” OR “smart phone” OR smartphone OR “cellular phone”) AND (addiction OR dependence OR abuse OR dependency OR “addicted to” OR overuse OR “problem use” OR “compensatory use”) AND (self-control OR self-regulation OR self-discipline OR effortful-control OR “impulse control”)

Limiters: Language – English

Timespan: All years

Boolean/Phrase: (“cell Phone” OR “mobile phone” OR “smart phone” OR smartphone OR “cellular phone”) AND (addiction OR dependence OR abuse OR dependency OR “addicted to” OR overuse OR “problem use” OR “compensatory use”) AND (“social support” OR “social care” OR “online social support” OR “perceived social support”)

Limiters: Language – English

Timespan: All years
