# Supplementary material for: The associations between smartphone addiction and self-esteem, self-control, and social support among Chinese adolescents: A meta-analysis
Source: Front Psychol. 2022 Nov 7;13:1029323. doi: 10.3389/fpsyg.2022.1029323 (PMC9677120; doi:10.3389/fpsyg.2022.1029323)
Supplement: Supplementary file 3 [file Table_3.DOCX]

Table S1.Quality assessment for the 56 studies in the current meta-analysis.

| Study | Quality Item | | | | | | | | | |
| --- | --- | --- | --- | --- | --- | --- | --- | --- | --- | --- |
|  | Item1 | Item2 | Item3 | Item4 | Item5 | Item6 | Item7 | Item8 | Item9 | Total |
| Wang(2011)(69) | Y | Y | Y | Y | Y | Y | Y | Y | Y | 9 |
| Yu(2013)(70) | Y | N | Y | Y | Y | Y | Y | Y | Y | 8 |
| Xu(2014)(71) | Y | N | Y | N | Y | Y | U | Y | Y | 6 |
| Deng(2015)(72) | Y | Y | Y | Y | Y | Y | N | Y | N | 7 |
| Li(2016)(42) | Y | Y | Y | Y | Y | Y | Y | Y | Y | 9 |
| Pan(2016)(73) | Y | U | Y | N | Y | Y | Y | Y | Y | 7 |
| Yang(2016)(74) | Y | U | Y | N | Y | Y | Y | Y | Y | 7 |
| Wang(2017)(75) | Y | U | Y | N | Y | N | Y | Y | Y | 6 |
| Li(2017)(76) | Y | Y | Y | N | Y | Y | Y | Y | Y | 8 |
| Liu(2017)(77) | Y | N | Y | Y | Y | Y | N | Y | Y | 7 |
| Jia(2018)(78) | Y | Y | Y | Y | Y | N | Y | Y | Y | 8 |
| Yu(2018)(79) | Y | N | Y | Y | Y | Y | Y | Y | Y | 8 |
| Liu(2018)(80) | Y | Y | Y | Y | Y | Y | Y | Y | Y | 9 |
| Wang(2018)(81) | Y | N | Y | N | Y | Y | Y | Y | Y | 7 |
| Liu(2018)(82) | Y | U | Y | Y | Y | N | Y | Y | Y | 7 |
| Duan(2018)(83) | Y | Y | Y | Y | Y | Y | Y | Y | Y | 9 |
| Wang(2018)(47) | Y | U | Y | N | Y | N | Y | Y | Y | 6 |
| Wang(2018)(84) | Y | N | Y | Y | Y | Y | Y | Y | Y | 8 |
| Zou(2018)(85) | Y | Y | Y | N | Y | Y | U | Y | Y | 7 |
| He(2019)(86) | Y | Y | Y | N | Y | N | U | Y | Y | 6 |
| Li(2019)(87) | Y | N | Y | Y | Y | N | Y | Y | Y | 7 |
| Xiang(2019)(23) | Y | Y | Y | Y | Y | Y | Y | Y | Y | 9 |
| Zheng(2019)(88) | Y | N | Y | Y | Y | Y | Y | Y | Y | 8 |
| Zhu(2019)(89) | Y | Y | Y | Y | Y | Y | Y | Y | Y | 9 |
| Gao(2019)(90) | Y | Y | Y | Y | Y | N | Y | Y | Y | 8 |
| Li(2019)(91) | Y | Y | Y | Y | Y | Y | Y | Y | Y | 9 |
| Wang(2019)(92) | Y | U | Y | Y | Y | N | Y | Y | Y | 7 |
| Gao(2020)(93) | Y | Y | Y | N | Y | Y | Y | Y | N | 7 |
| Peng(2020)(28) | Y | U | Y | Y | Y | N | Y | Y | Y | 7 |
| Li(2020)(94) | Y | U | Y | Y | Y | Y | N | Y | Y | 7 |
| Xing(2020)(95) | Y | N | Y | Y | Y | Y | Y | Y | Y | 8 |
| Huang(2020)(96) | Y | Y | Y | Y | Y | N | Y | Y | Y | 8 |
| Ma(2020)(97) | Y | Y | Y | Y | Y | Y | Y | Y | Y | 9 |
| Gao(2020)(98) | Y | U | Y | Y | Y | N | Y | Y | Y | 7 |
| Ma(2020)(99) | Y | Y | Y | Y | Y | N | Y | Y | Y | 8 |
| Niu(2020)(100) | Y | N | Y | Y | Y | Y | Y | Y | Y | 8 |
| Xiang(2020)(101) | Y | N | Y | Y | Y | Y | Y | Y | Y | 8 |
| Jiao(2020)(46) | Y | N | Y | Y | Y | Y | Y | Y | N | 7 |
| Fu(2020)(32) | Y | U | Y | Y | Y | N | Y | Y | Y | 7 |
| Hu(2021)(102) | Y | Y | Y | Y | Y | Y | Y | Y | Y | 9 |
| Liu(2021)(103) | Y | Y | Y | Y | Y | Y | Y | Y | Y | 9 |
| Yang(2021)(104) | Y | Y | Y | Y | Y | Y | Y | Y | Y | 9 |
| Kong(2021)(105) | Y | Y | Y | Y | Y | N | Y | Y | Y | 8 |
| Wang(2021)(39) | Y | U | Y | N | Y | Y | Y | Y | Y | 7 |
| Wang(2021)(106) | Y | N | Y | Y | Y | Y | Y | Y | Y | 8 |
| Zhao(2021)(107) | Y | N | Y | Y | Y | Y | Y | Y | Y | 8 |
| Li(2021)(31) | Y | Y | Y | Y | Y | N | Y | Y | Y | 8 |
| Cui(2021)(108) | Y | Y | Y | N | Y | N | Y | Y | N | 6 |
| Tian(2021)(109) | Y | Y | Y | Y | Y | N | Y | Y | Y | 8 |
| Zhang(2021)(110) | Y | Y | Y | Y | Y | Y | Y | Y | Y | 9 |
| Li(2022)(111) | Y | N | Y | Y | Y | N | Y | Y | Y | 7 |
| Chen(2022)(112) | Y | Y | Y | Y | Y | N | Y | Y | Y | 8 |
| Tian(2022)(113) | Y | U | Y | Y | Y | N | Y | Y | Y | 7 |
| Hu(2022)(114) | Y | Y | Y | Y | Y | Y | Y | Y | Y | 9 |
| Wang(2022)(115) | Y | Y | Y | Y | Y | N | Y | Y | Y | 8 |

*Abbreviations:* Y, yes; N, No; U, unclear.

Note: 1. Was the sample frame appropriate to address the target population?; 2. Were study participants sampled in an appropriate way?; 3. Was the sample size adequate?; 4. Were the study subjects and the setting described in detail?; 5. Was the data analysis conducted with sufficient coverage of the identified sample?; 6. Were valid methods used for the identification of the condition?; 7. Was the condition measured in a standard, reliable way for all participants?; 8. Was there appropriate statistical analysis?; 9. Was the response rate adequate, and if not, was the low response rate managed appropriately?
